# Retention and outcomes for clients attending a methadone clinic in a resource-constrained setting: a mixed methods prospective cohort study in Imphal, Northeast India

**DOI:** 10.1186/s12954-020-00413-z

**Published:** 2020-09-29

**Authors:** Michelle Kermode, Robedi Sharma Choudhurimayum, Lenin Singh Rajkumar, Tilahun Haregu, Greg Armstrong

**Affiliations:** 1 Nossal Institute for Global Health, University of Melbourne, Level 5/333 Exhibition St, Melbourne, VIC 3010 Australia; 2 Department of Psychiatry, Regional Institute of Medical Science, Imphal, Manipur India

**Keywords:** Methadone, Opioid substitution therapy, Drug dependence, Cohort study, India

## Abstract

**Background:**

Opioid substitution therapy (OST) with buprenorphine has been widely available in India since 2007, but the introduction of methadone occurred much later in 2012, and availability remains limited. Illicit injecting drug use is a long-standing public health problem in Manipur, a state in Northeast India characterised by major resource constraints and political unrest. We investigated retention and outcomes for clients attending a methadone-based OST program in Manipur with the aim of strengthening the evidence base for development of relevant policies and programs.

**Methods:**

All clients enrolling in the methadone clinic over a 1 year period were invited to be part of a prospective cohort study, which followed up and surveyed both retained and defaulting clients for 12 months post-enrollment to assess retention as well as social, behavioural and mental health outcomes. Additionally, we conducted semi-structured qualitative interviews to supplement quantitative information and identify factors contributing to retention and drop-out.

**Results:**

Of the 74 clients enrolled, 21 had dropped out and three had died (all defaulters) by 12 months post-enrollment, leaving 67.6% still in the program. Using an intention-to-treat analysis, meaningful and statistically significant gains were observed for all social, behavioural and mental health variables. Between baseline and 12 months there were reductions in needle sharing, drug use, property crime, anxiety, depression and suicidal thoughts; and improvements in physical health, mental health, quality of family relationships, employment and hopefulness. Factors contributing to retention and drop-out were identified, including the centrality of family, and general lack of awareness of and misunderstanding about methadone.

**Conclusion:**

Even in parts of India where resources are constrained, methadone is an effective treatment for opioid dependence. Scaling up the availability of methadone elsewhere in Manipur and in other areas of India experiencing problematic opioid dependence is indicated.

## Introduction

Opioid substitution therapy (OST) supplies people who inject drugs (PWID) with a medically prescribed replacement drug such as methadone or buprenorphine, which is usually administered orally in a supervised clinical setting. The benefits of OST are well-documented in a range of settings and include: reductions in illicit opiate use, HIV risk behaviours, death from overdose, criminal activity, and financial and other stresses for PWID and their families; as well as better adherence to antiretroviral therapy (ART) and improved physical and mental health [1–4]. According to UNAIDS, PWID are 29 times more likely to be infected with HIV compared to the general population [5], so a reduction in HIV risk behaviours is particularly advantageous for this group. The available evidence highlights that the longer a person is retained in an OST program, the greater the benefits [1], so understanding contextual factors that contribute to OST retention and drop-out is a strategy for strengthening the program and improving the benefits for PWID, their families and communities.

Despite the recognised benefits of OST, it is estimated that only 3% of PWID in India are currently receiving it [6], and lingering doubts about the wisdom of providing OST are widespread among government, health care providers and communities in some countries, including India [7–10]. Alongside the delivery of well-established harm reduction programs targeting PWID across the country, buprenorphine as OST was scaled up in India by the National AIDS Control Organization (NACO) beginning in 2007. However, the introduction of methadone is a more recent event, and challenges to scale-up remain despite its cheaper cost, positive findings from a pilot program implemented in five sites across the country [11], and evidence supporting the superiority of methadone compared to buprenorphine in terms of retention in OST [4]. Consequently, accessing buprenorphine in India is somewhat easier than methadone, and evidence to support buprenorphine in this context has been well-documented [12–15]. Providing policy makers in India with a similarly robust body of locally generated evidence regarding retention and outcomes for clients receiving methadone can contribute to the scaling up of a contextually appropriate methadone program in areas of high need, if findings lend support to this approach.

The site of our study was Manipur, a state of India located in the Northeast region, with a population of approximately 2.8 million. It shares a long porous border with Myanmar and due to illicit trafficking of heroin across the border, injecting drug use has been a major public health problem for around three decades. The prevalence of HIV in Manipur in 2017 was 1.17% among the general population, and 7.7% among PWID (reduced from 28.6% in 2008) [16, 17]. The local response to HIV is led by the Manipur State AIDS Control Society and includes the provision of OST (primarily buprenorphine) to PWID who meet certain criteria as defined by NACO i.e. diagnosis of opioid dependence with an injected drug; aged ≥ 18 years; previously failed detoxification; and able to give informed consent. Methadone as OST was introduced in Manipur in 2012 as part of a pilot program [11], but at the time of our study, which commenced in 2017, the program had not been scaled up, and methadone was available at one site only i.e. the Department of Psychiatry, Regional Institute of Medical Science (RIMS) in Imphal, the capital of Manipur. Manipur is a state with a long history of political unrest, which can cause major disruptions to the delivery of services including health care [18]. The society is structured around tightly knit communities that are often tribally and geographically based, and family life is central to people’s everyday lives [18]. Consequently, PWID, most of whom are male, tend to remain living in the family home, and therefore do not have to worry about access to food and shelter [18], unlike PWID in many other parts of the world.

The research reported in this paper is a mixed methods longitudinal prospective cohort study that followed people who were dependent on illicit injected opiates entering a newly established methadone-based OST program in a resource poor setting (Manipur, Northeast India) for a 1 year period. It is an example of operational research which is defined as ‘The search for knowledge on interventions, strategies, or tools that can enhance the quality, effectiveness, or coverage of programs in which the research is being done.’ [19]. The aim was to generate local evidence to strengthen the methadone program in Manipur, and if the outcomes were overall positive, for advocacy with government to scale-up the program elsewhere in India. The scaled-up availability of methadone as OST in India has the potential to contribute to a more affordable and effective national harm reduction program (as it has done in other countries), and thereby improve the health and well-being of PWID, their families and communities.

The specific objectives were to:

- Quantify retention rates for a methadone-based OST program
- Describe OST client and clinic staff perceptions of factors that promote retention or contribute to drop-out from methadone treatment
- Map health, social and behavioural outcomes in the year following registration in a methadone-based OST program
- Document perceptions of the service from the perspective of the service users.

## Method

We undertook a mixed methods, longitudinal, prospective cohort study of OST clients attending a clinic at the Integrated OST Centre, Department of Psychiatry, Regional Institute of Medical Science (RIMS) in Imphal, capital of the Northeast Indian state of Manipur. The method closely adhered to the protocol proposed by the WHO Collaborative Study on Substitution Therapy of Opioid Dependence and HIV/AIDS [20].

### Study participants and recruitment

The inclusion criteria for the study were:

- Eligible for registration with the OST program (as defined by NACO)
- Willing to participate in a longitudinal study and be interviewed over a 12 month period
- Willing to provide contact details for him/herself as well as family and/or friends in order to facilitate follow-up in the event of drop-out
- Able to give informed consent.

All newly registered OST program clients over a one-year period were invited to participate in the quantitative survey, which commenced in September 2016. Study participants were followed up for 1 year, so the period of survey data collection was completed in September 2018. Clients who left the OST program prematurely (whatever the reason) were followed up wherever possible i.e. when they were contactable and consented to participation. Purposive sampling was used to recruit participants for the semi-structured qualitative interviews to ensure that at least one-quarter were clients who had dropped out of the program.

### Data collection

Using an interviewer administered survey, we collected data pertaining to the following: socio-demographic characteristics; perceptions of the OST program; OST retention; drug use patterns and history; HIV risk behaviours; HIV testing; and mental health. The questionnaire was administered at commencement of treatment (baseline, within 2 weeks of commencing treatment), and 6 weeks, 6 months, and 12 months post enrolment. At each of these time points, questions regarding adherence, drug use, and risk behaviours were asked in relation to the previous 4 weeks, and for mental health, the previous 2 weeks.

Additionally, we conducted semi-structured qualitative interviews with OST clients and OST clinic staff (counsellors and nurses) with the aim of describing the health, social and behavioural impacts of OST on the lives of PWID, and the factors that promoted retention or contributed to drop-out from OST treatment. Findings from the qualitative interviews provide contextual information to supplement and enrich understanding of treatment benefits, retention and relapse, thereby strengthening usefulness of the findings. Qualitative interviews with OST clients were conducted between 2–4 months post-enrolment in the program in either Manipuri or English, audio-recorded and subsequently translated and transcribed.

Data collection was undertaken by two locally recruited male research officers who had many years of experience working with PWID in Manipur (one was a former PWID), and were specifically trained to collect data using the survey instrument and via semi-structured qualitative interviews. Data collection with clients retained in the program took place in a private location within the health facility. For clients who had left the program, data collection took place at a mutually agreed location that was comfortable and safe for both the PWID and the person collecting the data.

### Data collection tools

The survey questionnaire comprised: validated scales with strong psychometric properties, a sub-set of questions on injecting behaviours adapted from the Integrated Behavioural and Biological Assessment (IBBA) survey previously undertaken among high-risk groups in six states of India in 2009 [21], and some questions designed by the researchers. When developing the questionnaire we used/adapted the following validated scales:

1. Patient Health Questionnaire scale (PHQ-9)—a 9 item tool that screens for symptoms of depression [22], with clinically meaningful cut-off scores. The PHQ-9 has been validated for use in India [23], and scores range from 0 to 27 with a score of 10 or higher indicating moderate to severe depressive symptoms.
2. Generalised Anxiety Disorder scale (GAD-2)—a 2 item tool that screens for symptoms of anxiety [24], which has previously been used in India [25]. Scores range from 0 to 6 with a score of 3 or higher representing the optimum cut-off point to screen for anxiety disorders. The GAD-2 has high sensitivity (86%) and specificity (83%) for detecting generalised anxiety disorder [26].
3. Suicidality: Current suicidal ideation was assessed using Item-9 of the PHQ-9 [22], which asks participants how often they had ‘thoughts you would be better off dead or of hurting yourself in some way’. This measure was dichotomised to those participants who responded ‘several days’, ‘more than half the days’ and ‘nearly every day’ and those who responded ‘not at all’. Questions from the Suicide Behaviour Questionnaire (SBQ-R) were used to assess experiences of suicidal thoughts and attempts in the preceding 12 months [27].

The questions designed by the authors pertained to social inclusion (e.g. Do you generally participate in your family social events? Never, rarely, sometimes, always); quality of life (e.g. Do you feel hopeful for your future? Very hopeful, somewhat hopeful, not hopeful); and quality of the OST program (e.g. How easy is it for you to access the clinic each day? Very difficult, difficult, easy, very easy). All measurement instruments were translated and back-translated by bi-lingual members of the research team, and piloted in the field to ensure equivalence of meaning for the questions and response categories, and to ensure that the total questionnaire time did not exceed one hour. Copies of the baseline and follow-up survey questionnaires are available upon request from the first author.

The qualitative interviews with the OST clients and clinic staff were guided by a tailored theme list, the development of which was informed by the study objectives and the literature. It was translated and back-translated to ensure equivalence of meaning.

### Data analysis

Quantitative data were analyzed using Stata version 15.0. The socio-demographic, health and behavioral characteristics of the study population are presented in tables using descriptive statistics. Intention-to-treat analysis was used to assess changes in health, social and behavioural outcomes; we used follow-up data when it was available and replaced missing values with baseline data when follow-up data was missing. Thus, we conservatively assumed no change for clients who had dropped out and were not able to be followed-up. Cochran chi-square test was used to assess differences in the outcomes of interest across the four time points. We used McNemar’s test for two paired proportions as a post hoc (multiple comparison) test to compare outcomes between two time points (e.g. baseline to 12 months, 6-months to 12-months, etc.), to identify at what follow-up time points significant changes were first observed and then retained. We used Cohen’s h as a measure of effect size for the change in proportions between baseline and follow-up; h = 0.2 is "small", h = 0.5 is "medium", and h = 0.8 is "large" [28]. We also present the changes in health, social and behavioural outcomes for those retained in the program separately (excluding drop-outs) to highlight the magnitude of benefits for those who are able to remain in the program. All tests were two-tailed and *p* values < 0.05 were considered to be statistically significant.

Qualitative semi-structured interviews were audio-recorded, transcribed and translated by an experienced medical translator/transcriber. The completed interview transcripts were inductively and deductively thematically analysed [29]. All coding was done using OpenCode 4.03. Development of the codes was initially a deductive process, informed by the research questions, interview guides, relevant literature and field experience. These codes were subsequently refined and new codes identified through inductive interpretation of the data. All codes were subsequently grouped under relevant overarching themes in order to address the research questions. Quotes from the qualitative interviews are used where appropriate to bring life to the quantitative findings.

## Results

A total of 74 OST clients were enrolled in the cohort study during the 12 months of recruitment (only one client declined to participate). More than two-thirds (67.6%, n = 70) had previously been enrolled in OST (all buprenorphine) and subsequently relapsed. The majority (94.6%, 70/74) were prescribed methadone. Following their intake interview, four clients were referred for buprenorphine instead of methadone.

A total of 20 semi-structured qualitative interviews were conducted with OST clients (14 retained clients and 6 clients who had left the program; all were male), and seven interviews were conducted with OST staff (4 counsellors, 2 nurses, 1 doctor). Data from these interviews are used to supplement and enrich quantitative findings where appropriate.

### Socio-demographic characteristics of survey participants

The mean age of OST clients was 33.3 years (median 31; range 18–58), and most were male (94.6%). More than half (58%) had been married (of whom six were either separated, widowed or divorced), and 54.1% had children. The level of literacy was 96.0%, which was relatively high for India, which according to 2011 Census of India was 74%. Similarly, previous school attendance was high by Indian standards; 48.7% had completed schooling, and 23.0% had gone on to attend college or university (Table 1). There were no statistically significant differences in these socio-demographic characteristics between those retained in the OST program and those who dropped out during the follow-up period.

**Table 1:** Baseline socio-demographic and behavioral characteristics of study participants (n = 74)

Participant characteristics	Number	Percent
Sex		
Male	70	94.6
Female	4	5.4
Age (in years)		
18–30	31	41.9
31–58	43	58.1
Marital status		
Ever married	43	58.1
Never married	31	41.9
Have children		
No	34	46.0
Yes	40	54.1
Can read and write		
Yes	71	96.0
No	3	4.1
Highest level of education		
Never attended school	4	5.4
Completed 5–11 years	34	46.0
Completed class 12	19	25.7
Completed college/university	17	23.0
Employed		
No	32	43.2
Yes, full-time	32	43.2
Yes, part-time	10	13.5
Main source/s of income (multiple responses allowed)		
Family/friends	60	81.1
Employment	26	35.1
Others (e.g. pension, house rent etc.)	10	13.5
Participation in family social events		
Never	10	13.5
Rarely	14	18.9
Some of the time	22	29.7
Always	28	37.8
Quality of family relationships		
Good	2	2.7
Fair	19	25.7
Poor	53	71.6
Physical health at baseline (self-perceived)		
Fair, good or very good	19	25.7
Poor	55	74.3
Drugs used in the last 12 months (multiple responses allowed)		
Heroin	72	97.3
Benzodiazepines	53	71.6
ATS—amphetamine-type-stimulants	38	51.4
Cannabis/ganja	34	46.0
Spasmoproxyvon^a^	29	39.2
Cough syrup	12	16.2
Raw opium	4	5.4
Brown sugar	2	2.7
Antihistamines	1	1.4
Others	4	5.4
Drug injected the most in last 12 months		
Heroin	72	98.6
Other	1	1.4
Frequency of use of most injected drug (past 4 weeks)		
Once a day or less	4	5.4
2–3 times a day	21	28.4
More than 3 times a day	48	64.9
Needle sharing (past 4 weeks)		
Yes	37	50.0
No	37	50.0
Ever been arrested by police		
Yes	60	81.1
No	14	18.9
Number of times arrested by police (if arrested)		
Once	17	23.0
More than once	43	58.1
Ever been in prison		
Yes	9	12.2
No	65	87.8
Property crime (past 4 weeks)		
No	32	43.2
Yes	42	56.8
Alcohol use (past 4 weeks)		
Never consumed alcohol	41	56.2
Not in the past month	5	6.9
Less than once a week	16	21.9
At least once a week	9	12.3
Every day	2	2.7
Depression symptoms^b^		
No (PHQ-9: < 10)	19	25.7
Yes (PHQ-9: ≥ 10)	55	74.3
Anxiety symptoms^c^		
No (GAD-2 < 3)	44	59.5
Yes (GAD-2 ≥ 3)	30	40.5
Suicidal thoughts (past 12 months)		
No	19	25.7
Yes	55	74.3
Suicide attempt (past 12 months)		
No	55	76.4
Yes	17	23.6
Current suicidal ideation (past 2 weeks)^d^		
No	30	40.5
Yes	44	59.5
Ever had an HIV test		
No	7	9.5
Yes	67	90.5
Ever been tested for HCV		
Yes	51	68.9
No	23	31.1

^a^A synthetic opioid analgesic containing dextropropoxyphene

^b^Patient Health Questionnaire (PHQ-9) scores of ≥ 10 represent the optimum cut-off when screening for depression

^c^Generalised Anxiety Disorder (GAD-2) score of ≥ 3 represents the optimum cut-off when screening for anxiety

^d^Assessed using Item 9 of the PHQ-9

### Retention and drop-out

Of the 74 clients enrolled, 20 had dropped out and two had died by 6 months post-enrollment, leaving 70.3% still in the program; one more client had dropped out and one more had died by 12 months post enrollment, leaving 67.6% still in the program at completion of the study. The three clients who died had dropped out of the program. These retention rates indicate that the risk of drop out is greatest during the first 6 months of the program (Table 2).

**Table 2:** Retention in the program

	Number	Percent
6 weeks		
Retained	71	95.9
Dropped out *(cumulative total)*	3	4.1
Total	74	100.0
6 months		
Retained	52	70.3
Dropped out^a^ *(cumulative total)*	22	29.7
Total	74	100.0
12 months		
Retained	50	67.6
Dropped out^a^ *(cumulative total)*	24	32.5
Total	74	100.0

We were able to obtain follow-up data from some clients who had dropped out at 6 weeks (n = 2), 6 months (n = 10) and 12 months (n = 7)

^a^Includes three deaths of people who had already dropped out of the program; two by 6 months and an additional one between 6 and 12 months

Factors identified by qualitative interview participants as supportive of retention included: the inability to get pleasure from drug use while taking methadone; being married and having children; family support, monitoring and encouragement; increased feelings of self-respect and hopefulness for the future; gradually losing the desire for drugs; and valuing the reduction in tension associated with obtaining money and drugs, overdosing, and avoiding police. Quotes illustrating each of the factors contributing to retention can be found in Table 3. According to participants, the greatest challenges to adherence were encountered in the earlier stages of the program; once they had stabilized on treatment and time passed without relapse, a new life gradually emerged to replace the old one.

**Table 3:** Quotes illustrating factors contributing to retention in the OST program

Being married and having children	
*As I told you earlier the bachelors don’t think about their future. If they were married they would obviously think about their kids and the future. When I was a bachelor I didn’t care about anything other than my drugs. A meal for me at home is taken for granted, so I don’t care about the future or earning. So my lifestyle when I was a bachelor and today after I am married is very much different… In those days, I only thought about taking the drugs and nothing about the future at all. But today, even when I want to take the drugs, I cannot take them after thinking about my wife and children. I think it is because of this that bachelors don’t think about the future, so they are not compelled to quit the drugs. They don’t have to think about a wife neither do they care about earning any money.* ***Participant 6 (dropped out)***	

Less tension and more hopefulness	
*The most important thing is that it saves me a lot of money. There is no tension for buying the drugs, and relationships with family members is also becoming better. I can also feel the changes in how people look at me. There is no tension for money, for getting arrested by the police, and I don’t have to go at North AOC [drug using hotspot] anymore. Now I want to dress up smartly and hope to buy a two-wheeler vehicle of my own. If fact, I have many dreams for my future which were not there at all earlier. ****Participant 18 (retained)***	
*There were times when I used to stop taking drugs, and at those times I suffered a lot and had a very hard life. I even thought of committing suicide. And so instead of living that fearful life, I feel it is better to stay on methadone. When I am on methadone I never have that kind of feeling, never have those kind of negative thoughts. As long as I am on methadone there is no suffering in my life.* ***Participant 4 (retained)***	

Family support	
*One point is that most of the clients who are staying in the program for a long time, their family members are quite supportive. They are monitored closely by their family members, even when they are going to miss one dose, they [the family members] will call up and communicate with the counsellor and service provider. So that kind of relationship and communication is necessary with the family member and service provider. They would also ask if he has gone to take his dose for today… There is need for good support.* ***Service provider 25***	
*When I was taking drugs, then no one would support me as they are now. There would be no one to give me even 5 rupees at that time. Now, if I go for taking the methadone, they will give me money to get the fuel for the four-wheeler instead of the two-wheeler. On top of that they will ask me if I have some pocket money. Sometimes when there is a shortage of vehicles for me to go to the centre, they will arrange a vehicle for me somehow by postponing another task, with the thought that going to the centre to take the methadone is very much necessary.* ***Participant 13 (retained)***	
*When my mother, father or wife support me, like when they wake me up they will remind me about the medication. If I don’t have the fare to go at the centre, they will manage somehow and give it to me. So these kinds of things give me some encouragement. Further, when I see them peeking and waiting for me to return home and getting worried that I may go the wrong way again, I understand their feelings and think I will not go back to that way again.* ***Participant 5 (retained)***	
*Yes, it is because of the support from the family members that people can come here daily to take the methadone. My home is a little far away from the clinic, but I can come here daily because of my family support. Whether there is any strike or bandh, I used to reach the clinic anyhow with my family support and never missed my dose.* ***Participant 16 (retained)***	
*Without the family’s support, I don’t think a person could continue on the program successfully. Initially I used to come here for taking the methadone along with my family, my wife or uncle. At the initial stage when the dose was small, I got wearied and blew out, so at this stage if a person is alone he might have gone in a wrong direction. So I feel that family support is very much necessary here.* ***Participant 19 (retained)***	
*(I)f the family members give proper support, there is less chance of drop out. If the family support is less, the chance of drop out is high. At the time of induction phase, we inform them that they (family members) can always come along with the client, but this is mandatory for 2 months’ minimum. So, I would say that most of the drop outs are due to lack of family support.* ***Service Provider 27***	

Factors (both hypothetical and actual) identified by qualitative interview participants as contributing to drop-out included: wanting to experience the pleasure of drug use again; the influence of friends who were still using drugs; side-effects of treatment; absence of family support; family conflict; accident or illness; inconvenient hours of the clinic; misunderstanding/misinformation about methadone; pressure from family members to end treatment prematurely; and wanting a faster pathway to a drug-free life. Quotes illustrating each of the factors contributing to drop-out can be found in Table 4. Some of the clients who dropped out subsequently entered a rehabilitation facility in order to reach a point of abstinence more rapidly, but tended to relapse once discharged from the rehabilitation centre.

**Table 4:** Quotes illustrating factors contributing to drop-out from the OST program

Wanting to experience the pleasure of drugs	
*In my view, yes, taking methadone keeps us away from the pain, but it does not give us any pleasure. After taking the methadone, people become free from all the tension about money or police, but sometimes people like to enjoy the pleasure of the drugs which they don’t get it if they take the methadone. Even for me, during the initial stage of starting the methadone, I had an extreme desire to enjoy the pleasure of drugs just for once… So I think the chief reason for dropout would be people having the desire to enjoy the pleasure of taking the drugs.* ***Participant 18 (retained)***	
*He [the client] can’t forget the pleasure that drugs give, and when he sees someone experiencing the pleasure of the drugs, he also wants to experience again that pleasure he once had. With methadone there is no kick or pleasure, it only gives relief from the enduring pain… He thinks of continuing the drugs for some more time as the methadone opportunity will always be there down the road.* ***Participant 4 (retained)***	
*I started mixing up the methadone with other drugs and enjoyed the pleasure, which continued further. Later on, a problem cropped up with the family members regarding money and other issues, so they finally put me in the rehabilitation centre.* ***Participant 20 (dropped-out)***	

Influence of drug using peers	
*It might be that there are still some active users in his locality who persuade him, or he cannot set apart from them. After taking the methadone dose he may have returned to a group of active users who are still his friends, even though he hasn’t taken drugs for some days. When he mingles with these active users, he could feel the odd man out…. So this could be one of the reasons.* ***Participant 4 (retained)***	

Wanting a faster pathway to a drug-free life	
*I joined the program thinking that it was some kind of detoxification and hoping it would work for me. I thought I could be free from drugs in a short duration of time, but it happened to be long term, so I left the program. I had wanted to quit the drugs very soon, but as the methadone program consists of one year, I could not continue that long. So I did not complete the program.* ***Participant 6 (dropped-out)***	
*For me I wanted the total abstinence. I did not want to continue depending on anything else. I wanted to be free from everything, so I dropped out from the program… I never had any problem with the methadone. Taking the methadone was far better than taking the drugs. I only wanted a total abstinence without depending on any other drugs.* ***Participant 9 (dropped out)***	

Misunderstanding/misinformation about methadone	
*Interviewer: Have you ever thought of quitting the methadone?Respondent: Yes, sometimes I had that feeling. But who flees from a tiger only to confront a bear… Even though the methadone is not exactly like the heroin, it is still some kind of drug. So there is a feeling that if we continue taking it for long time, it might be harmful for us. There is an apprehension of being dependent on the methadone.* ***Participant 14 (retained)***	
*I had heard that methadone is very harmful to the body and wherever the methadone flows inside the body, it gets damaged. After that I thought of quitting it and once I asked the counsellor if there are any side effects with the methadone. The counsellor told me there is no side effect except some constipation and slight effect to the teeth. They told me not to worry and I got more relaxed after hearing that. ****Participant 18 (retained)***	

Pressure from family members	
*There are some situations where the clients have dropped out due to the influence of the parents because the parents thought that as their son has taken methadone for a few months and has changed, it would be better for him to quit the methadone rather than depending on it for a longer period of time. Hoping so, their parents withdraw their son from the program and their son gets relapsed after a few days.* ***Service provider 26***	

### Benefits of retention in the OST program

Table 5 presents an intention to treat analysis of the benefits of retention in the OST program for the entire cohort, excluding those who had died. Major gains were made in relation to a number of important variables when baseline is compared with the 6 weeks follow-up data; and while the benefits have attenuated somewhat when baseline is compared with the 12 months follow-up data, the gains remain impressive and statistically significant for all variables. Despite the relatively small sample size, between baseline and 12 months there was significant and meaningful reductions in the levels of needle sharing and drug use over the last 4 weeks, property crime, anxiety, depression, and suicidal thoughts. There were significant and meaningful improvements in physical health, mental health, the quality of family relationships, participation in family events, employment and hopefulness for the future. In order to demonstrate the benefits of OST for those who were retained in the program, Table 6 presents the same outcomes as Table 5 excluding those who had dropped out.

**Table 5:** Intention to treat analysis examining impact of OST participation on social and behavioral outcomes (with baseline replacement for lost to follow up)

	Baseline (T0)	6 weeks (T1)	6 months (T2)	12 months (T3)	*P* value^a^	T0–T1		T0–T2		T0–T3	
	n (%)	n (%)	n (%)	n (%)		Effect size^b^	*P* value^c^	Effect size^b^	*P* value^c^	Effect size^b^	*P* value^c^
Injecting drug use (past 4 weeks)	73 (98.7)	7 (9.5)	19 (25.7)	20 (27.0)	0.000	2.286	0.000	1.850	0.000	1.820	0.000
Shared needle for injection (past 4 weeks)	37 (50)	3 (4.1)	10 (13.5)	10 (13.5)	0.000	1.163	0.000	0.818	0.000	0.818	0.000
Property crime (past 4 weeks)	42 (56.8)	3 (4.1)	13 (17.6)	12 (16.2)	0.000	1.299	0.000	0.841	0.000	0.879	0.000
Anxiety symptoms (GAD-2: ≥ 3)^d^	30 (40.5)	3 (4.05)	9 (12.2)	11 (14.9)	0.000	0.975	0.000	0.667	0.000	0.588	0.000
Depression symptoms (PHQ-9: ≥ 10)^e^	55 (74.3)	4 (5.4)	22 (29.7)	16 (21.6)	0.000	1.609	0.000	0.926	0.000	1.112	0.000
Current suicidal ideation (PHQ-9: item 9)^f^	44 (59.5)	12 (16.2)	19 (25.7)	22 (29.7)	0.000	0.933	0.000	0.699	0.000	0.609	0.000
Poor physical health (self-perceived)	55 (74.3)	4 (5.4)	10 (13.5)	14 (18.9)	0.000	1.609	0.000	1.326	0.000	1.179	0.000
Poor family relationships	53 (71.6)	2 (2.7)	17 (23)	16 (21.6)	0.000	1.687	0.000	1.017	0.000	1.051	0.000
Very hopeful for future	9 (12.2)	16 (21.6)	28 (37.8)	32 (43.2)	0.000	0.253	0.127	0.611	0.000	0.721	0.000
Employment (employed)	42 (56.8)	38 (52.8)	43 (60.6)	53 (71.6)	0.007	0.080	0.439	0.077	0.832	0.310	0.016
Participation in family events	50 (67.6)	51 (68.9)	57 (77)	60 (81.1)	0.079	0.028	0.847	0.211	0.189	0.312	0.018

^a^Cochran chi square test for equality of proportions

^b^Effect size was based on Cohen’s h (h = 0.2 is “small”, h = 0.5 is “medium”, and h = 0.8 is “large”)

^c^McNemar’s test for the difference between two proportions

^d^Generalised Anxiety Disorder (GAD-2) score of ≥ 3 represents the optimum cut-off when screening for anxiety

^e^Patient Health Questionnaire (PHQ-9) scores of ≥ 10 represent the optimum cut-off when screening depression symptoms

^f^Assessed using Item 9 of the PHQ-9

**Table 6:** Impact of OST participation on social and behavioral outcomes among those retained at T1, T2 and T3 (excluding drop-outs)

	Baseline (T0) (n = 74)	6 weeks (T1) (n = 71)	6 months (T2) (n = 52)	12 months (T3) (n = 50)	*P value*^*a*^	T0–T1		T0–T2		T0–T3	
	n (%)	n (%)	n (%)	n (%)		*Effect size*^*b*^	*P value*^*c*^	*Effect size*^*b*^	*P value*^*c*^	*Effect size*^*b*^	*P value*^*c*^
Injecting drug use (past 4 weeks)	73 (98.7)	6 (8.1)	7 (9.5)	3 (4.1)	0.000	2.336	0.000	2.286	0.000	2.505	1.000
Shared needle for injection (past 4 weeks)	37 (50)	1 (1.4)	0 (0)	0 (0)	0.000	1.334	0.000	1.571	0.000	1.571	0.000
Property crime (past 4 weeks)	42 (56.8)	1 (1.4)	0 (0)	0 (0)	0.000	1.470	0.000	1.707	0.000	1.707	0.000
Anxiety symptoms (GAD-2: ≥ 3)^d^	30 (40.5)	2 (2.82)	1 (1.9)	2 (4)	0.000	1.042	0.000	1.103	0.000	0.977	0.000
Depression symptoms (PHQ-9: ≥ 10)^e^	55 (74.3)	2 (2.8)	5 (9.6)	0 (0)	0.000	1.742	0.000	1.448	0.000	2.078	0.000
Current suicidal ideation (PHQ-9: item 9)^f^	44 (59.5)	10 (14.1)	7 (13.5)	9 (18)	0.000	0.992	0.000	1.009	0.000	0.886	0.000
Poor physical health (self-perceived)	55 (74.3)	3 (4.1)	2 (3.2)	1 (1.8)	0.000	1.671	0.000	1.719	1.000	1.809	0.000
Poor family relationships	53 (71.6)	1 (1.8)	7 (11.3)	3 (5.3)	0.000	1.748	0.000	1.332	1.000	1.553	0.000
Very hopeful for future	9 (12.2)	16 (22.5)	28 (53.9)	31 (62)	0.000	− 0.275	0.189	− 0.935	0.000	− 1.100	0.000
Employment (employed)	42 (56.8)	36 (52.2)	33 (67.4)	40 (80)	0.010	0.092	0.607	− 0.219	0.648	− 0.507	0.019
Participation in family events	50 (67.6)	50 (70.4)	44 (84.6)	44 (88)	0.019	− 0.061	0.845	− 0.405	0.210	− 0.504	0.039

^a^Cochran chi square test for equality of proportions

^b^Effect size was based on Cohen’s h

^c^McNemar’s test for the difference between two proportions

^d^Generalised Anxiety Disorder (GAD-2) score of ≥ 3 represents the optimum cut-off when screening for anxiety

^e^Patient Health Questionnaire (PHQ-9) scores of ≥ 10 represent the optimum cut-off when screening depression symptoms

^f^Assessed using Item 9 of the PHQ-9

Many qualitative interview participants described a range of life changing benefits that they attributed to participation in the OST program including more harmonious family relationships, greater stability, improved self-esteem, better health, and reduced craving for drugs, as illustrated in the following quotes:

Positively, there have been many changes. I have a good relationship with my spouse and other family members which I didn’t have earlier. I can go for my work properly which I could not do before. There has been improvement in my body and my health. I am restored to my previous life as it was when I was not taking any kind of drugs. I have changed completely from the situation when I used to think only about drugs for 24 h every day… I can perform my duty in a proper manner and in proper time, whether it be house work, duty, personal work, helping my kids in their studies, or anything else. Earlier I used to forget everything—that I have work, duty, wife, and children. So I want to say I am having this much improvement. ***Participant 13 (retained)***

While I was taking the drugs, there were many crimes or offensive activities that I performed with the family members and in neighbouring areas. Telling lies to other people just to get the drugs, extracting things from other people and cheating, mostly just to get the drugs. But now, after I am on methadone, this thing never comes to my mind and I never think of telling a lie. The methadone kick deflects us from thinking such things. ***Participant 5 (retained)***

I am very much happy with the OST program because my life now is much more stable than the earlier life. I can now perform my duties well, like a normal person. The outcome of the methadone is really good for me as I started using the drugs right from an early stage of my life. I have not really experienced how a normal life is. It is only now that I have experienced it. ***Participant 11 (retained)***

### Drug and alcohol use

As can be seen in Table 1 multiple drug use at baseline was commonly reported, with heroin being the primary drug injected during the preceding 12 months for 98.6% of the cohort. The median duration of time since first ever injection of illicit drugs was 7 years (range 1–37 years, IQR 3–16 years). Most clients (94.6%) had been injecting at least 2–3 times per day during the previous 4 weeks. Alcohol use was less common (Manipur is technically a dry state); 63.1% had not used any alcohol during the previous 4 weeks, and only 2.7% had used alcohol on a daily basis.

At baseline 98.7% reported injecting drugs in the preceding 4 weeks, which had reduced to 27.0% by 12 months post-enrollment (only 4.1% among those still enrolled). At baseline, 50.0% had shared needles during the previous 4 weeks, which had decreased to 13.5% by 12 months post-enrollment (0% among those who were retained) (Tables 5 and 6).

### Employment

Current or previous occupations included police, army, shopkeeper, small businessman, government, airlines, farming and students. The main sources of income at baseline were family and friends for 81.1% of clients, and employment for 35.1%. At baseline, 56.8% of clients were employed. By 12 months post-enrollment, 71.6% were employed (80.0% amongst those retained) (Tables 5 and 6).

Those participants who were employed whilst on OST acknowledged that having access to methadone improved their work performance.

The difference is while I was taking drugs I couldn’t do anything properly or completely, let alone my work. Before my work was completed, I would give some silly excuse and escape. But while I was taking the methadone I could perform my duties completely in fulltime from 9am to 4 pm—that is full office hours. ***Participant 25 (dropped out)***

Some qualitative interview participants reported that their family members did not want them to work until they had been stabilized on treatment for an extended period of time as they believed that having access to money would increase the likelihood of relapse, while other participants said that they wanted to focus on remaining adherent to methadone for a year or so before actually looking for work. These perspectives are reflected in the following quotes:

My family thinks that if I start earning and have cash with me, maybe I will return to my earlier stage as they have already seen repeatedly. They feel that I should not start earning, but also have a plan that I should start earning later on. ***Participant 5 (retained)***

Firstly, my most important plan is just to complete my one year program and see if I can cope with the recovery. Thereafter, I will see if I can get a job… or even return to my former job. ***Participant 4 (retained)***

### Family relationships and social inclusion/exclusion

The quality of family relationships was poor for 71.6% of clients at baseline, which had reduced to 21.6% by 12 months post-enrollment (only 5.3% among those who were retained). Significant gains were also evident in relation to increased participation in family events, with 67.6% participating in family events at baseline, which had increased to 81.1% at 12 months (88% among those who were retained) (Tables 5 and 6).

Many of the qualitative interview participants described poor family relationships prior to joining the OST program, as is evident in the following quotes:

While I was on drugs there were frequent fights at home and I committed many unwanted things like selling our home appliances. Once my mother had some money for our father’s treatment as he was not well at that time. I had a knife in my hand and snatched the money from my mother’s hands by force. ***Participant 9 (dropped-out)***

About family relationships—before I was on methadone, when I asked for money from my parents, they used to ask me so many questions and want to know for what and where I was going to use it, although they still gave it to me. I would give them any silly reason, but then they easily knew that I was telling a lie just to get the money…. but still I used to take it anyhow, which made them feel upset and disturbed. I too felt sad and disturbed but I would take the money anyway and go to the hotspot area. ***Participant 4 (retained)***

Participation in the OST program was often associated with substantial improvements in family relationships, especially in relation to gradual re-establishment of trust, reduction in conflict, and inclusion in family events and decision making. These gains are well described in the following quotes:

Someone may have tried various other OST programs but did not succeed. They may have also stayed in rehabilitation centres many times and their family members had completely lost trust in them. For such clients, methadone is very helpful… we have seen it… There are some clients here whose family members totally avoided them and did not trust them at all, but after taking the methadone they have changed completely and have started trusting them again now. ***Service provider 26***

There have been lots of changes even from the family member’s side too. They have started listening to me properly and they have started trusting me a little. They know that I am no longer taking the drugs because of the change that they see now, compared to the earlier times when I was taking the drugs. So it makes me feel happy and proud that they now have started discussing the family matters with me. ***Participant 14 (retained)***

A few participants reported that some family members remained distrustful and did not support their participation in the methadone program.

There are some family members who do not understand the methadone program, so when he [the client] comes back after taking the methadone, they scold him thinking he has taken the drugs. It also happened with me the same when I was taking only the OST for about 6 to 7 months. A family member thinks that I have returned home after taking the drugs. ***Participant 10 (dropped out)***

Every family doesn’t necessarily have the same feeling or same understanding [of the OST program] and some have bitter neighbours. Neighbouring people who know very little about OST, backbite and spread rumours saying that he is going to RIMS [clinic] to take OST or some medication because he was using drugs earlier, making his parents ashamed of him. Furthermore, it makes the family members scold him for his deeds, and they urge him to stop going to RIMS as it has no benefit. Instead they are being insulted and getting so many complaints from the local people. So he must have stopped coming because the family has been unsupportive. ***Participant 4 (retained)***

Clients sometimes asked counsellors to make contact with their families to clarify misunderstandings about the methadone program, and some family members would contact counsellors to make sure the client was attending, and to check on their progress. In this cultural context, where medical consultations are often with families rather than individuals, these linkages between staff, clients and their family members were generally perceived to have therapeutic value among both clients and staff.

Social exclusion prior to enrolment in the OST program, both initiated by and imposed upon the participant, was well described during qualitative interviews, as was greater social inclusion post-enrollment in the program. This is clearly reflected in the following quotes:

Before I was on methadone, my thinking and feeling was like I was a sick person. I could not perform any kind of work, and there was even lack of connection with my surroundings and society. I could not even attend any ceremony or occasion being held in my neighbourhood. I felt weak and felt depressed or anxious before using methadone, but after using methadone I feel normal. The negative feelings and inability to attend social activities is now not there at all. ***Participant 4 (retained)***.

When I was taking the drug, I usually tried to avoid my friends as much as possible. Even if they came and asked me to join them for some outing, I usually ignored them by saying I have some other work, because I have to think how to get the drugs for that day. ***Participant 22 (retained)***

When I was using the drugs my friends ignored me when I went to them so I got frustrated and mingled with the drug users. Now they know and have noticed that I gave up the drugs so they started accepting me as a friend… So now the friendship is getting started little by little, which was totally gone away earlier. ***Participant 18 (retained)***

### Physical and mental health

At baseline, 74.3% of clients reported poor physical health, which decreased to 18.9% by 12 months post-enrollment (1.8% among those who were retained). Most (90.5%) had previously had an HIV test, and 68.9%% had previously had a test for HCV. A majority (59.5%) had an HIV test and 39.2% had an HCV test at least once during the 12 months post-enrollment. Improved physical health was mentioned during qualitative interviews, especially in relation to energy levels, weight gain and appetite. This was attributed to improved lifestyle as well as being able to access good quality medical care at RIMS, including treatment for HIV and HCV infection.

At baseline, 74.3% of clients screened as potentially depressed, and 40.5% as having an anxiety disorder. This had decreased to 21.6% depressed and 14.9% anxious by 12 months post-enrollment (0% depressed and 4% anxious among those who were retained). Initially, only 12.2% of clients felt very hopeful about their future; this increased to 43.2% (62.0% among those who were retained) by 12 months post-enrollment (Tables 5 and 6). At baseline, 74.3% reported experiencing suicidal thoughts in the last year, and 23.6% had actually attempted suicide. By 12 months, only 18.0% reported suicidal ideation in the last year.

During qualitative interviews several participants described depressed and suicidal feelings prior to enrollment in the OST program.

Suicidal thoughts came into my mind because I had suffered a lot and could not do anything… Sometimes I felt guilty and didn’t want to take money from my parents everyday, and realised that what I was doing was not a good thing, so I thought of quitting [life] rather than endure the pain… Thereafter, I started thinking many fearful things like how is my life going to be, I will not be able to quit the drugs, I have given enough burden to my family, I am useless, I better leave the world. ***Participant 4 (retained)***

R: I have even tried committing suicide… While using the drugs, I was full of sadness and sorrow. I was just totally preoccupied.

I: How many times have you thought of committing suicide while you were on the drugs?

R: It was many a times. It was mostly when I don’t get the drugs… There arises a thought that am I going to remain like this forever and am unable to quit the drugs, so I should commit suicide. At home, while I am on drugs, parents and other family members kept scolding me. While using the drugs family members hate me. They did many good things for me, but I kept on continuing it. ***Participant 10 (dropped out)***

### Encounters with the justice system

At baseline, many of the clients reported committing crimes and had encounters with the justice system. Three-quarters (81.1%) had been arrested by the police; of these, 58.1% had been arrested more than once. One in ten (12.2%) had been to prison, and 56.8% said they had committed property crime during the 4 weeks prior to enrolment in the program. Property crime had reduced to 16.2% by 12 months post-enrollment (0% among those retained in the program).

When I was taking the drugs, there was always apprehension about getting arrested by the police while buying the drugs or while injecting the drugs. When we got caught, the police snatched away the drugs as well as whatever money we had in our pocket… So this kind of stretch and strain is not there anymore. ***Participant 18 (retained)***

### Satisfaction with the OST program

Survey participants (excluding those who could not be followed up) were asked about their satisfaction with the OST program. The vast majority of participants (including those who had dropped out and were followed up) remained overall satisfied or very satisfied with the service and the staff (100% at 6 weeks and 98.2% at 12 months). A small proportion of clients (5.5% at 6 weeks and 7.0% at 12 months) found accessing the clinic every day difficult or very difficult, and a similar proportion found the clinic hours to be inconvenient (2.7% at 6 weeks and 3.5% at 12 months). A small proportion (5.5% at 6 weeks and 7.0% at 12 months) reported their withdrawal symptoms as somewhat controlled, rather than well controlled. During qualitative interviews, the service and staff were consistently praised. However, a few participants were of the view that the service needed to open earlier for the benefit of those working as daily wage labourers who often have to commence work earlier in the day. Some also acknowledged the need to make methadone available in other parts of the state, not only the state capital.

Qualitative interview participants (both clients and staff) proffered a range of suggestions for strengthening the program. Many expressed a need for targeted activities to raise awareness and understanding of methadone among PWID, families, NGOs and the broader community.

It [the methadone clinic] is just a rumour… people just hear from one another. So I suggest that there should be some kind of advertisement or publicity about it so that ignorant people come to know about it, and can get the benefit from the centre. It is better to take the methadone rather than taking the drugs. ***Participant 6 (dropped out)***

Most importantly, I would like all drug users to join this program. There still must be many who are not aware of this program. As the staff of this program, please reach out to as many people as you can, and I will also let as many people know about this in addition to bringing my friends… Please conduct the program as decently as it is now, and hopefully more people will get enrolled. ***Participant 11 (retained)***

The aim of such activities would be to make people (PWID especially) aware of the programs existence, nature and purpose, and to counteract misinformation regarding methadone in particular. Several participants said they would like to be able to access take-away doses when having to travel for work purposes or family events or when sick, suggesting that parents or spouses could collect and administer the dose on these occasions. The need for support groups for clients and families, and for community outreach to clients and families in their homes were also mentioned.

## Discussion

This prospective mixed methods cohort study of clients attending a methadone-based OST program in the resource constrained setting of Manipur, Northeast India, reported a high retention rate comparable with those found in other countries, and delivered impressive health, social and behavioural outcomes, especially for those who were retained in the program longer-term. To the best of our knowledge, this is the first prospective cohort study of methadone clients in India. Additionally, the inclusion of clients who dropped-out, and the use of mixed methods strengthen the findings and enrich understanding of the lived experience of OST clients.

The previous methadone pilot study conducted in five sites across India reported 60.5% retention at 6 months and 35.9% at 12 months [11], which is less than the 70.3% at 6 months and 67.6% at 12 months observed in this study. Internationally, a large study of OST programs in multiple countries, reported an average retention at 6 months of 70% (based on retention in eight countries), which was as low as 55% in Australia, and as high as 88% in China [1].

While retention levels were very good, recruitment of new clients was not as high as initially anticipated, which contributed to the relatively small sample size. Many of the study participants mentioned a need to raise awareness of methadone as OST treatment in order to inform PWID, their families and communities about the program, and to dispel prevalent myths about the safety and efficacy of methadone. Awareness raising among health care workers and NGOs providing services for PWID is also indicated as they are likely to be a major source of client referrals to the methadone clinic.

Similar to previous studies [1–4], clients who remained in the program experienced significant benefits across the 12 month follow-up period including: marked reductions in drug use, HIV risk behaviours and property crime; along with major improvements in physical and mental health, family relationships, social inclusion and employment. The benefits of this OST program are likely to extend beyond the clients to include their families and communities.

The multiple benefits accrued by the clients who remained in the OST program highlights the need to actively maximize retention. Close monitoring of clients during the first few months when the risk of drop-out is much greater, and active follow-up of those whose attendance is dropping off (much as those who are lost-to-follow up in TB treatment programs are actively followed up) may promote greater retention.

The poor mental health and quality of life of the PWID clients at baseline is concerning. Not only were high levels of probable depression and anxiety evident, but suicidal ideation and suicidal attempts were commonplace. A similarly high prevalence of psychological distress and suicidal ideation and attempts were reported among clients attending needle syringe programs in New Delhi [25, 30, 31], accompanied by calls to integrate mental health and suicide prevention programs into harm reduction programs for PWID [32]. It was encouraging to note significant improvements in the mental health of the clients who remained in the program, which is consistent with evaluations of OST elsewhere in the world [33, 34].

It is worth noting the extent to which the OST service in Manipur is family focused rather than (individual) person focused. This was particularly evident in the qualitative interviews. The majority of clients were living with their family members, dependent on them for all of their daily needs, and very aware of the negative impact their drug dependence was having on family life. It is the view of the authors that a family-centred approach to care is essential in a society where the family unit and local community confer identity, and where limited social welfare is available. Family involvement was seen as a strength by both clients and service providers, and therefore considered necessary for successful recovery. Other authors reporting from non-Western countries such as China and Timor Leste have similarly acknowledged the need for a family-centred rather than client-centred approach to mental health and addiction services [35, 36]. The role of the family in drug treatment in China is considered to be more important relative to more individualistic western cultures, due to the family-orientation of traditional Chinese culture. Consequently, in the field of drug-dependence treatment, families in China play an essential role in encouraging PWID to initiate and remain in treatment; and similar to the situation in Manipur, most PWID in China stay with their families before and after attending treatment programs [35].

A number of study limitations should be considered when interpreting these findings. Firstly, the design of this applied operational research did not include a control group. However, given what is already known about the efficacy of methadone from studies outside of India, the inclusion of a control group would not be ethically justifiable. Additionally, the sample size was relatively small—originally we anticipated enrolling 150 clients over a 12 month period, but uptake of the program was not that high. This has resulted in the inferential analyses being underpowered. Further, the small sample size and the small number of dropouts from whom data could be collected meant we were unable to compare outcomes for those who have dropped out with those who were retained. The lower enrolment than originally anticipated in the methadone-based OST program may in part be due to the fact that clients had to reside within a 5 km radius of the clinic. As the treatment is a directly observed therapy, clients have to be able to reach the clinic by foot at times of political unrest when vehicles are prohibited. While the findings are very pertinent to this specific context, due to the above limitations, they should be generalized with caution. Most PWID in Manipur are familiar with buprenorphine-based OST clinics situated in NGOs that offer a range of harm reduction services for PWID including social programs. OST clinics based in a hospital setting may be less inviting for some PWID. However, none of the clients we interviewed were troubled by accessing their OST in a hospital setting. Another possible limitation is the fact that those who had dropped out and were willing to complete the survey or participate in the qualitative interviews were likely to be quite different from those who had dropped out but could not be followed up. The intention to treat analysis for the survey data compensates for this limitation to some extent, but this was not the case for the qualitative interviews. The interview transcripts were translated from Manipuri to English. As if often the case with language translation, direct word-for-word translation was not always possible, which compromises the qualitative researcher’s capacity to accurately detect nuance and idiosyncratic meanings of words in context.

## Conclusion

The findings from this prospective mixed methods cohort study of clients enrolled in a methadone-based OST program located in a resource-constrained setting reported relatively high retention of clients; major improvements in HIV risk behaviours, mental health and social well-being; and high levels of client satisfaction with the service and the staff (even among those who had dropped out). Scaling up the availability of methadone elsewhere in Manipur and other areas of India where opioid dependence is problematic is indicated, as is concurrent awareness raising about methadone to increase knowledge and dispel myths among PWID, their families and communities, NGOs and health care providers.

## Data Availability

The datasets generated and analysed during the current study are not publically available because study participants did not give explicit consent for the raw data to be made publically available (except via de-identified selected quotes). The datasets are available from the corresponding author on reasonable request.
